# Increased circulating polymorphonuclear myeloid-derived suppressor cells are associated with prognosis of metastatic castration-resistant prostate cancer

**DOI:** 10.3389/fimmu.2024.1372771

**Published:** 2024-06-03

**Authors:** Takuro Kobayashi, Masayoshi Nagata, Tsuyoshi Hachiya, Haruhiko Wakita, Yoshihiro Ikehata, Keiji Takahashi, Toshiyuki China, Fumitaka Shimizu, Jun Lu, Yiming Jin, Yan Lu, Hisamitsu Ide, Shigeo Horie

**Affiliations:** ^1^ Department of Urology, Graduate School of Medicine, Juntendo University, Tokyo, Japan; ^2^ Department of Advanced Informatics for Genetic Diseases, Graduate School of Medicine, Juntendo University, Tokyo, Japan

**Keywords:** castration-resistant prostate cancer, hormone-sensitive prostate cancer, myeloid-derived suppressor cell, prognosis, tumor microenvironment

## Abstract

**Introduction:**

Myeloid-derived suppressor cell (MDSC) exhibits immunosuppressive functions and affects cancer progression, but its relationship with prostate cancer remains unclear. We elucidated the association of polymorphonuclear MDSC (PMN-MDSC) and monocytic MDSC (M-MDSC) levels of the total peripheral blood mononuclear cells (PBMCs) with prostate cancer progression and evaluated their roles as prognostic indicators.

**Methods:**

We enrolled 115 patients with non-metastatic hormone-sensitive prostate cancer (nmHSPC, n = 62), metastatic hormone-sensitive prostate cancer (mHSPC, n = 23), and metastatic castration-resistant prostate cancer (mCRPC, n = 30). Subsequently, the proportions of MDSCs in each disease progression were compared. Log-rank tests and multivariate Cox regression analyses were performed to ascertain the associations of overall survival.

**Results:**

The patients with mCRPC had significantly higher PMN-MDSC percentage than those with nmHSPC and mHSPC (P = 7.73 × 10^−5^ and 0.0014). Significantly elevated M-MDSC levels were observed in mCRPC patients aged <70 years (P = 0.016) and with a body mass index (BMI) <25 kg/m^2^ (P = 0.043). The high PMN-MDSC group had notably shorter median survival duration (159 days) than the low PMN-MDSC group (768 days, log-rank P = 0.018). In the multivariate analysis including age, BMI, and MDSC subset, PMN-MDSC was significantly associated with prognosis (hazard ratios, 3.48; 95% confidence interval: 1.05–11.56, P = 0.042).

**Discussion:**

PMN-MDSC levels are significantly associated with mCRPC prognosis. Additionally, we highlight the remarkable associations of age and BMI with M-MDSC levels in mCRPC, offering novel insights into MDSC dynamics in prostate cancer progression.

## 1 Introduction

Prostate cancer is the second most frequently diagnosed cancer and the fifth leading cause of cancer-related mortality worldwide. The incidence of prostate cancer was estimated to range from 1 to 4 million cases per year in 2020 and is projected to nearly double to between 2 to 9 million cases annually by 2040 (1). The number of deaths due to prostate cancer was 375,000 in 2020 and is estimated to increase by 85%, reaching nearly 700,000 by 2040 (1). Prostate cancer is a predominantly diagnosed cancer in 112 countries and is the primary cause of cancer death in 48 countries (2). The incidence and mortality rates of prostate cancer are positively associated with advancing age, with 66 years being the average age at diagnosis (3). Androgen deprivation therapy (ADT) is the primary treatment for cancer with advanced stages, but its effectiveness wanes over time. Many patients progress to castration-resistant prostate cancer (CRPC) within 2–3 years, which considerably worsens their prognosis (4, 5).

Recent insights into prostate cancer progression have spotlighted the role of myeloid-derived suppressor cells (MDSCs) within the tumor microenvironment (TME). MDSCs are a heterogeneous group of immature myeloid cells that exhibit immunosuppressive functions affecting various immune cells, and humans have two primary MDSC subtypes, which are as follows: polymorphonuclear MDSC (PMN-MDSC) and monocytic MDSC (M-MDSC) (6, 7). These cells, by expanding and activating within the TME, create an immunosuppressive environment that promotes cancer development by undermining innate and adaptive immune responses (8). In humans, MDSCs produce immunosuppressive cytokines, including TGF-β, IL-10, arginase 1, PGE2 (9, 10), stimulating regulatory T cells (11). High MDSC concentrations have been linked to unfavorable outcomes in various cancers (12–14). The microenvironment of prostate cancer varies due to the differences in hormone sensitivity; however, not all aspects have been fully elucidated (15). Therefore, clarifying the contribution of MDSCs is crucial for understanding the mechanism behind the acquisition of castration resistance in prostate cancer.

Several human studies on prostate cancer and MDSC subtypes have been conducted. A previous study reported elevated M-MDSC levels in patients with CRPC as compared with those in a healthy group (16). Another study on M-MDSC and prognosis in patients with CRPC reported that increased M-MDSC was associated with a poor prognosis (17). A previous survival analysis involving mCRPC patients showed that patients without elevated M-MDSC level after treatment had prolonged overall survival (OS) (18). Contrarily, the PMN-MDSC levels in prostate cancer patients correlated with advanced cancer stages and predicted poorer outcomes (19). A recent study on mHSPC patients indicated that PMN-MDSC is a negative prognostic indicator, whereas M-MDSC seemed to have no significant impact (20). Notably, no detailed studies have examined the background factors associated with MDSC subtype levels by classifying the prostate cancer patients according to hormone sensitivity and metastasis. Moreover, research comparing the prognostic value of the two MDSC subtypes in mCRPC patients is lacking. A recent meta-analysis explored the prognostic impact of circulating MDSC levels in patients with prostate cancer, and reported that those with high circulating MDSC levels had poorer prognosis as compared to those with lower MDSC levels (21). However, notable inconsistencies exist in defining the cutoff value across studies, with some studies employing methods such as median or mean while others use techniques such as Cox regression. This lack of standardization complicates the effective comparison of results among studies. Furthermore, as some studies did not identify the MDSC subtypes while others focused solely on M-MDSCs or PMN-MDSC, the absence of MDSC subtypes identification remains as a challenge (16–19, 21, 22). This inconsistency in reporting hampers the comprehensive understanding of the roles and impacts of MDSC.

The present research aimed to assess the association between MDSC subtypes and prostate cancer progression, considering hormone sensitivity and metastasis. Additionally, we also sought to evaluate the association of MDSC subtypes with prostate cancer prognosis.

## 2 Materials and methods

### 2.1 Patients and data collection

Patients with prostate cancer who provided consent to participate in this study from August 2019 and March 2023 at Juntendo University (Tokyo, Japan) were included. Patients with normalized prostate specific antigen (PSA) levels after 3 months of ADT with a GnRH antagonist (degarelix) or untreated patients were diagnosed with mHSPC and included in the study. CRPC was defined by a castrate serum testosterone level of <50 ng/dl or 1.7 nmol/l, along with either three consecutive PSA increases occurring at least 1 week apart resulting in at least two ≥50% increases over the nadir, with a PSA level of ≥2.0 ng/ml, or the appearance of new lesions on radiologic imaging (23). The patients who lacked prostate cancer activity and MDSC data and those with nmCRPC were excluded from the analysis.

For each patient, data on their age and body mass index (BMI), presented as mean ± standard deviation, were gathered. Given the nonparametric nature of the initial PSA (iPSA) levels, they were expressed as medians along with their respective ranges. For subsequent analysis, the patients were categorized into two groups with age of 70 years as the cutoff, BMI of 25 kg/m^2^ as the cutoff, and iPSA of 20 ng/mL as the cutoff. Additionally, we used the Gleason scoring system to assess the invasiveness of prostate cancer, classifying the patients into two groups based on scores of ≤7 and ≥8.

The assessment of the presence of metastases encompassed various sites, including bone, distant lymph nodes, and lung/liver/other sites during blood collection. Additionally, each patient’s treatment history, other than ADT, prior to blood sampling was recorded. This included a variety of treatments, including radical prostatectomy; radiation therapy [radium-223 and intensity modulated radiation therapy (IMRT)], heavy particle radiation, and postoperative salvage; androgen receptor axis targeted therapy (enzalutamide, abiraterone, apalutamide, and darolutamide); and chemotherapy, including docetaxel and cabazitaxel.

The study was conducted in accordance with the Declaration of Helsinki, and approved by the Institutional Review Board of the Juntendo University Institutional Review Board (protocol code: M19–0158 and H20–0187, date of approval: Nov. 1, 2019 and Sep. 11, 2020). Written informed consent was obtained from the patients to publish this paper.

### 2.2 MDSC measurement

**Figure 1:**
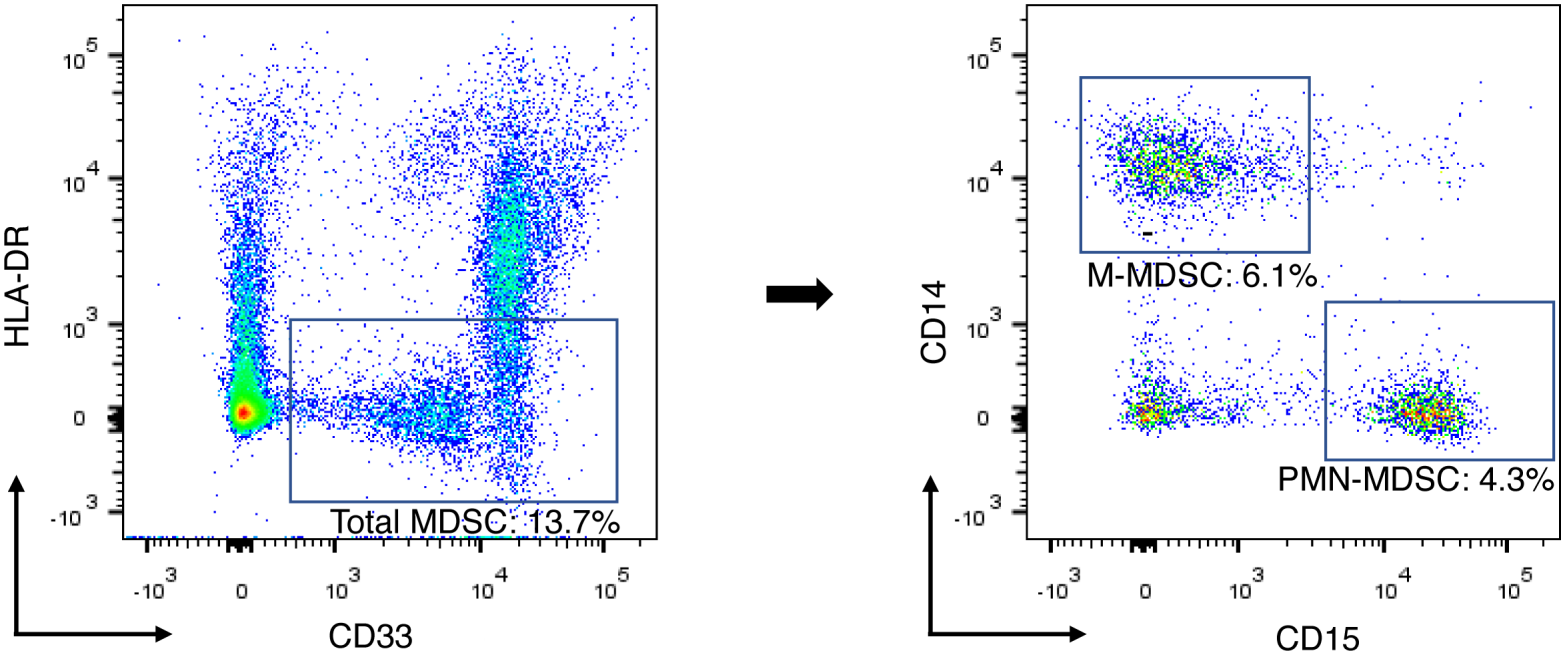
Gating strategy for identification of MDSC subsets and example of flow cytometry data. Total-MDSC, PMN-MDSC, and M-MDSC were characterized as HLA-DR^low/-^ CD33^+^, HLA-DR^low/-^ CD33^+^ CD15^+^ CD14^-^, and HLA-DR^low/-^ CD33^+^ CD15^-^ CD14^+^, respectively. Each population of the MDSC subsets was presented as a percentage of the total PBMCs. M-MDSC, monocytic myeloid-derived suppressor cell; PBMC, peripheral blood mononuclear cell; PMN-MDSC, polymorphonuclear myeloid-derived suppressor cell.

MDSCs were detected from fresh peripheral blood mononuclear cells (PBMC), isolated from peripheral blood by density gradient centrifugation using Histopaque^®^-1077 (Sigma-Aldrich, Missouri, United States). PMN-MDSCs are particularly sensitive to cryopreservation; thus, the assays of MDSC were performed using fresh samples immediately on the day of sample collection (24, 25). Altogether, 1.0 × 10^6^ single cells were suspended in 100-μl PBS and incubated with a FcR blocking reagent (Biolegend, California, United States) for 15 minutes at a room temperature, followed by an appropriate concentration of fluorescent-conjugated antibody in 100-μl PBS for 15 minutes at 4°C. PMN-MDSC and M-MDSC were characterized as HLA-DR^low/-^ CD33^+^ CD15^+^ CD14^-^ and HLA-DR^low/-^ CD33^+^ CD15^-^ CD14^+^ as a percentage of live cells in the total PBMC, respectively. The fluorochrome-labeled antibodies used for detecting cell surface antigens were CD14-PerCP-Cy5.5, CD15-APC-Cy7, CD33-PE-Cy7, and HLA-DR-PE-Texas Red (Biolegend, California, United States). The labeled cells were washed twice and resuspended in 500-μL buffer with DAPI (1 μg/mL). FACS data were acquired using the BD^®^ LSR II Flow Cytometer (BD Biosciences, California, United States) with BD FACSDiva™ software and analyzed using Flowjo software (Tree Star Incs, Oregon, United States). The gating strategy for MDSC is presented in **Figure 1**.

### 2.3 Statistical analysis

The missing values were handled by excluding cases with any missing data in our analysis. This approach resulted in the use of a complete-case dataset for all statistical analyses. The proportions of M-MDSC and PMN-MDSC, which are MDSC subtypes, were treated as nonparametric data. We compared these proportions using Mann–Whitney’s U test to explore their relationship with the patients’ characteristics, including hormone sensitivity and cancer metastasis. This analysis aimed to understand relation of the M-MDSC and PMN-MDSC levels with cancer progression, particularly in the context of hormone sensitivity and metastasis. For prognostic association analysis, M-MDSC and PMN-MDSC values were divided into two groups using the Youden index (sensitivity + specificity − 1): M-MDSC_low/high_ and PMN-MDSC_low/high_.

The Kaplan–Meier method was used to assess OS for all patients. Log-rank tests were used to compare the survival curves by prostate cancer progression and MDSC level. Additionally, hazard ratios (HRs) were estimated using the Cox proportional hazards model, with all confidence intervals (CI) at the 95% level. In addition to the univariate analysis, multiple models were created to adjust for potential confounders. In Model 1, age was treated as a category and adjusted for. Model 2 was adjusted for BMI as a category in addition to Model 1. Model 3 was further adjusted for the presence of bone metastases and chemotherapy effects, in addition to model 2. For further sensitivity analysis, Model 3 was adjusted for potential confounding between M-MDSC and PMN-MDSC.

For all analyses, a two-tailed P value of <0.05 was considered statistically significant. When comparing three groups, a corrected P value accounting for multiple comparisons (P = 0.05/3) was deemed statistically significant. All statistical analyses were performed using the R language, version 4.3.0 (R Foundation for Statistical Computing, Vienna, Austria).

## 3 Results

### 3.1 Patient characteristics

**Figure 2:**
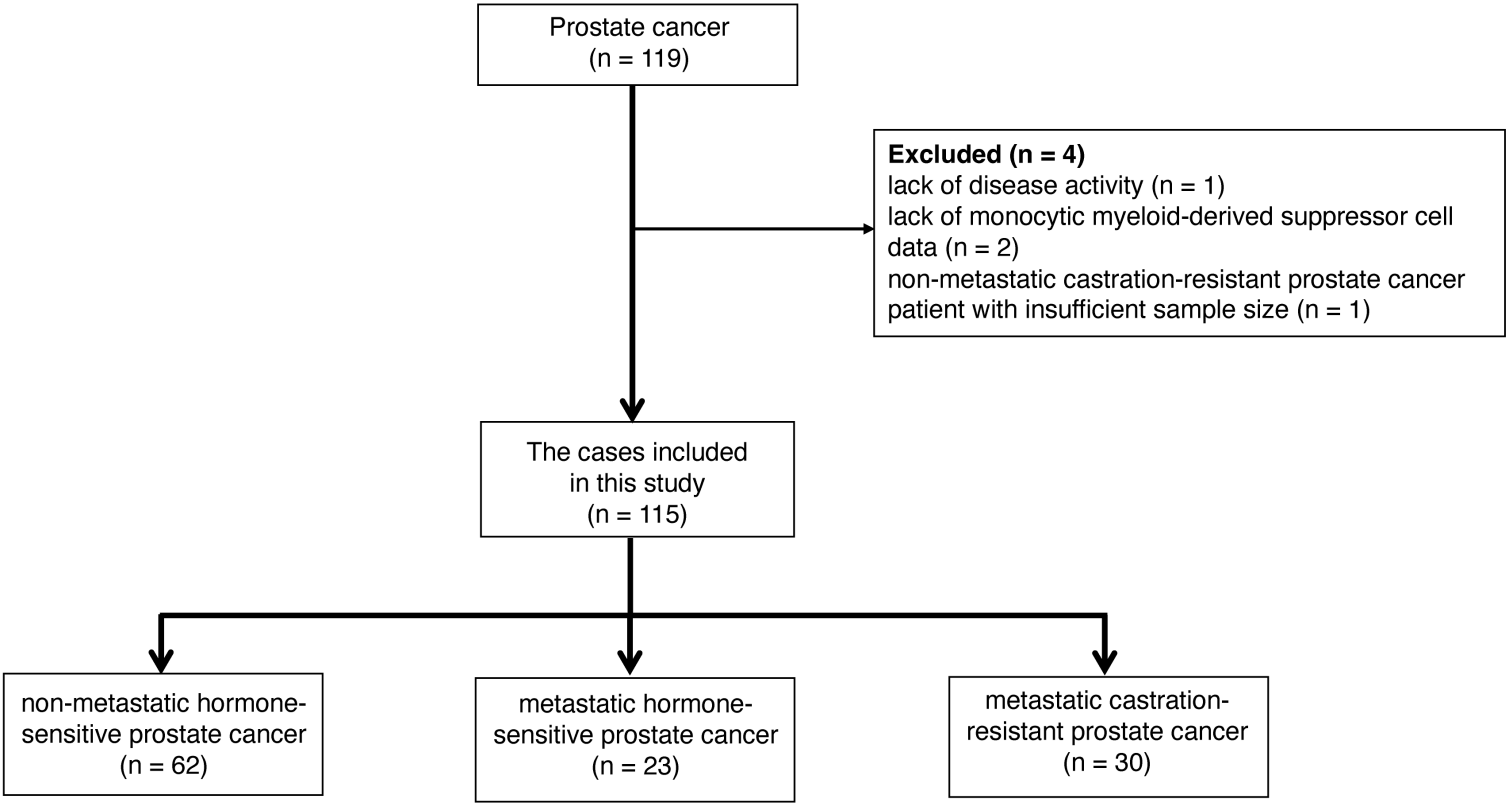
Strategy for selecting the study participants.

During the study period, 119 prostate cancer patients were assessed (**Figure 2**). One patient could not be evaluated due to indeterminate disease status, two were unable to provide MDSC data, and one patient with non-metastatic CRPC was excluded due to insufficient data for analysis. After applying the exclusion criteria, the final patient cohort comprised of 115 individuals, who were categorized in non-metastatic hormone-sensitive prostate cancer (nmHSPC, n = 62), metastatic hormone-sensitive prostate cancer (mHSPC, n = 23), and metastatic castration-resistant prostate cancer (mCRPC, n = 30) groups. The detailed demographic and clinical characteristics of these patients are presented in **Table 1**.

**Table 1:** Patients’ baseline characteristics.

Variables	nmHSPC (n = 62)	mHSPC (n = 23)	mCRPC (n = 30)
Age, years, mean ± SD	70.1 ± 7.7	70.2 ± 6.7	70.8 ± 9.4
<70, n(%)	28 (45.2)	7 (30.4)	11 (36.7)
≥70, n(%)	34 (54.8)	16 (69.6)	19
BMI, kg/m^2^, mean ± SD	24.1 ± 3.1	23.9 ± 3.5	22.2 ± 2.2
<25, n (%)	44 (71.0)	7 (30.4)	24 (80.0)
≥25, n (%)	18 (29.0)	16 (69.6)	5 (20.0)
iPSA, ng/mL, median(range)	8.8 (3.4–57.1)	107.4 (8.6–3025.0)	53.0 (2.8–9556.8)
<20, n(%)	53 (85.5)	3 (13.0)	11 (36.7)
≥20, n(%)	8 (12.9)	19 (82.6)	18 (60.0)
Gleason score			
<8, n(%)	43 (69.4)	2 (8.7)	7 (23.3)
≥8, n(%)	19 (30.6)	21 (91.3)	22 (73.3)
Metastatic sites			
Bone, n (%)	–	21 (91.3)	26 (86.7)
Distant lymph nodes, n (%)	–	6 (26.1)	11 (36.7)
Lung/Liver/Others, n (%)	–	9 (39.1)	10 (33.3)
Prior additional treatments to ADT			
Radical prostatectomy, n (%)	0 (0.0)	0 (0.0)	8 (26.7)
Radiation, n (%)	1 (1.6)	1 (4.3)	14 (46.7)
ARAT, n (%)	0 (0.0)	2 (8.7)	25 (83.3)
Chemotherapy, n (%)	0 (0.0)	0 (0.0)	12 (40.0)
None, n (%)	61 (98.4)	16 (69.6)	0 (0.0)

ADT, androgen deprivation therapy; ARAT, Androgen Receptor Axis Targeted; BMI, Body Mass Index; SD, standard deviation; iPSA, initial prostate specific antigen; mCRPC, metastatic castration-resistant prostate cancer; mHSPC, metastatic hormone-sensitive prostate cancer; nmHSPC, non-metastatic hormone-sensitive prostate cancer; SD, standard deviation.

### 3.2 MDSC levels and prostate cancer progression

**Figure 3:**
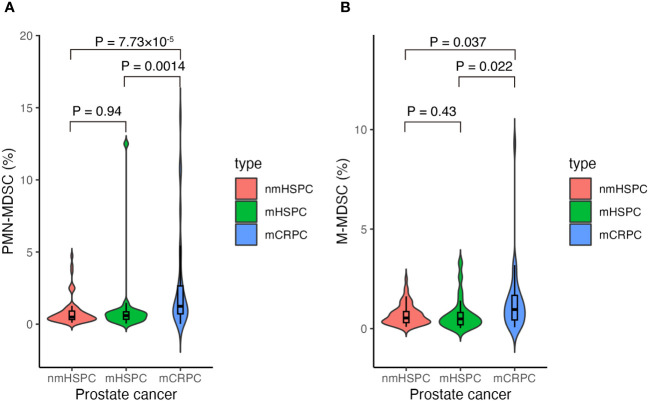
Violin and box plots of the percentage of MDSCs according to prostate cancer type. **(A)** PMN-MDSCs and prostate cancer. **(B)** M-MDSCs and prostate cancer. The x-axis shows prostate cancer disease status, whereas the y-axis shows the percentage of MDSCs in the total PBMCs. Differences were considered significant when p <0.05. mCRPC, metastatic castration-resistant prostate cancer; mHSPC, metastatic hormone-sensitive prostate cancer; nmHSPC, non-metastatic hormone-sensitive prostate cancer; M-MDSC, monocytic myeloid-derived suppressor cell; PBMC, peripheral blood mononuclear cell; PMN-MDSC, polymorphonuclear myeloid-derived suppressor cell.

We investigated the distribution of MDSC percentages in total PBMCs across different disease states. Notably, the median PMN-MDSC percentages were 0.50% (0.10%–4.73%) for nmHSPC, 0.59% (0.04%–12.50%) for mHSPC, and 1.24% (0.02%–14.40%) for mCRPC, revealing a significant increase in mCRPC (**Figure 3A**). These findings suggest that the MDSC levels may correlate with disease aggressiveness, particularly evident from the significant differences observed between nmHSPC and mCRPC (P = 7.73 × 10**^−^** ^5^) and between mHSPC and mCRPC (P = 0.0014).

The median values for M-MDSCs demonstrated an upward trend with disease progression; however, statistical significance was not maintained after adjusting for multiple testing (i.e., P > 0.05/3), highlighting the requirement for further investigation into their role across prostate cancer stages (**Figure 3B**).

### 3.3 Factors impacting the MDSC levels

**Figure 4:**
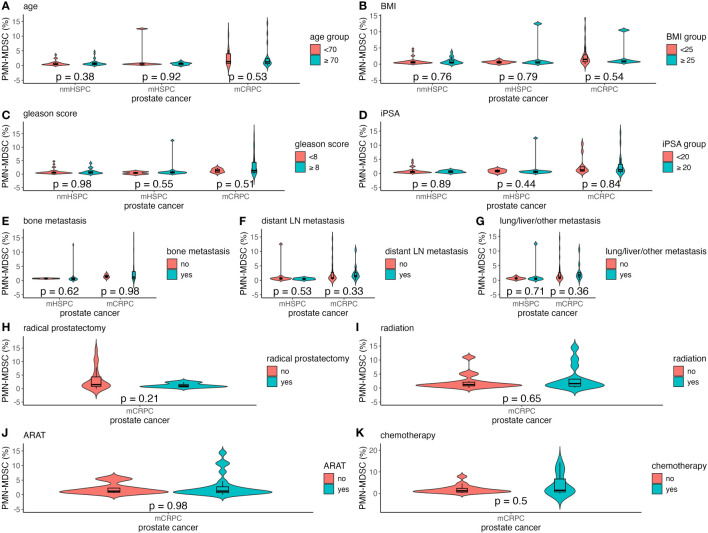
Violin and box plots of the percentage of PMN-MDSC by each factor in prostate cancer. **(A)** Age. **(B)** BMI. **(C)** Gleason score. **(D)** iPSA. **(E)** Bone metastases. **(F)** Distant lymph node metastases. **(G)** Lung/liver/other metastases. **(H)** Previous radical prostatectomy. **(I)** Previous radiation therapy. **(J)** Previous ARAT treatment. **(K)** Previous chemotherapy treatment. The x-axis indicates the comparison of prostate cancer disease status in the three groups, whereas the y-axis indicates the percentage of MDSCs in the total PBMCs. Differences were considered significant when p <0.05 within the respective figures. LN, lymph node; mCRPC, metastatic castration-resistant prostate cancer; mHSPC, metastatic hormone-sensitive prostate cancer; nmHSPC, non-metastatic hormone-sensitive prostate cancer; PBMC, peripheral blood mononuclear cell; PMN-MDSC, polymorphonuclear myeloid-derived suppressor cell.

**Figure 5:**
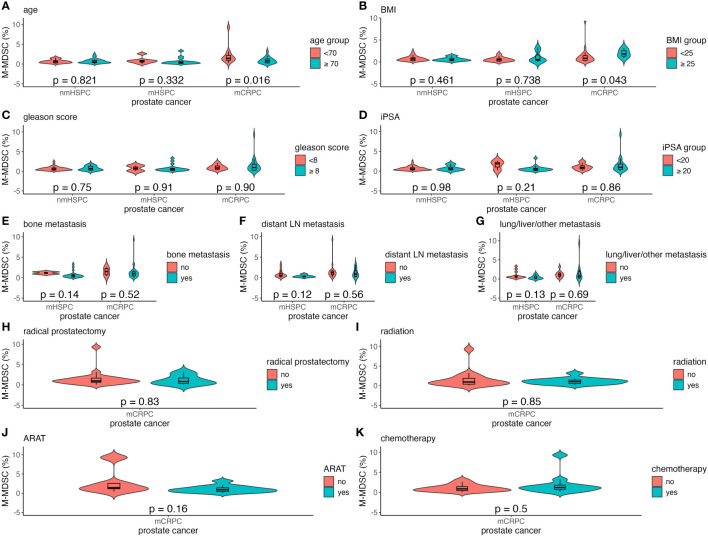
Violin and box plots of the percentage of M-MDSC by each factor in prostate cancer. **(A)** Age. **(B)** BMI. **(C)** Gleason score. **(D)** iPSA. **(E)** Bone metastases. **(F)** Distant lymph node metastases. **(G)** Lung/liver/other metastases. **(H)** Previous radical prostatectomy. **(I)** Previous radiation therapy. **(J)** Previous ARAT treatment. **(K)** Previous chemotherapy treatment. The x-axis indicates the comparison of prostate cancer disease status in the three groups, whereas the y-axis indicates the percentage of MDSCs in PBMCs. Differences were considered significant when p <0.05 within the respective figures. LN, lymph node; mCRPC, metastatic castration-resistant prostate cancer; mHSPC, metastatic hormone-sensitive prostate cancer; nmHSPC, non-metastatic hormone-sensitive prostate cancer; M-MDSC, monocytic myeloid-derived suppressor cell; PBMC, peripheral blood mononuclear cell.

Factors such as age, BMI, Gleason score, and iPSA were analyzed to understand their impact on MDSC percentages across the different prostate cancer stages (**Figures 4A–D**, **5A–D**). The relationship between metastasis and MDSC was exclusively explored in the mHSPC and mCRPC groups (**Figures 4E–G**, **5E–G**). Furthermore, the association between additional treatment history and MDSC was solely assessed within the mCRPC group (**Figures 4H–K**, **5H–K**).

Within the mCRPC group, younger patients showed a significantly higher M-MDSC percentage, with those aged <70 years having a median value of 1.43% (0.23%–9.29%), as compared to 0.77% (0.07%–3.19%) for those aged ≥70 years (P = 0.016) (**Figure 5A**). Additionally, a higher BMI was correlated with increased M-MDSC levels, with median values of 0.83% (0.07%–9.29%) and 1.81% (0.90%–3.19%) for BMI of <25 and ≥25 kg/m², respectively (P = 0.043) (**Figure 5B**). These associations were not observed in the nmHSPC and mHSPC groups.

### 3.4 Survival outcomes and MDSC levels

**Figure 6:**
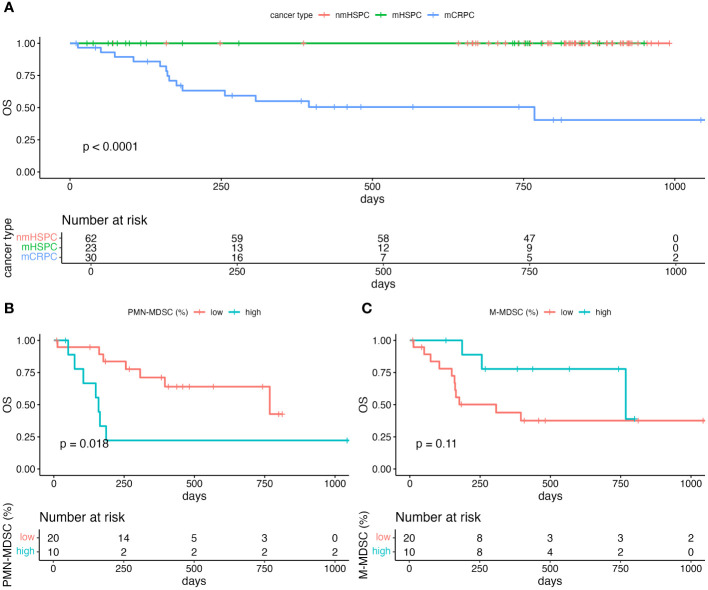
Kaplan–Meier curve of overall survival. **(A)** Comparison of the survival in each of the prostate cancer pathologies. Only survivors were observed in nmHSPC and mHSPC. **(B)** Comparison of the survival among mCRPC patients stratified by the level of PMN-MDSC. **(C)** Comparison of the survival among mCRPC patients stratified by the level of M-MDSC. Statistical significance of the survival distribution was analyzed by log-rank testing. mCRPC, metastatic castration-resistant prostate cancer; mHSPC, metastatic hormone-sensitive prostate cancer; nmHSPC, non-metastatic hormone-sensitive prostate cancer; PMN-MDSC, polymorphonuclear myeloid-derived suppressor cell; OS, overall survival.

The median survival durations for the nmHSPC and mHSPC group were 834 (71–994) and 732 (28–949) days, respectively. Conversely, the mCRPC group displayed a notably shorter median survival duration of 262 (10–1059) days. Throughout the observation period, no mortality events were noted in either the nmHSPC or mHSPC group. However, in the mCRPC group, 15 deaths were confirmed out of the 30 patients (**Figure 6A**). Given these observations, further analysis was undertaken, focusing on the PMN-MDSC and M-MDSC levels in the patients with mCRPC.

Using the Youden index to identify the optimal threshold for the area under the curve (AUC) of MDSC, the PMN-MDSC percentage of 2.135% showed a sensitivity of 86.7% and a specificity of 53.3% (**Supplementary Figure 1**). Conversely, the M-MDSC percentage of 1.365% presented a sensitivity of 46.7% and a specificity of 80.0% (**Supplementary Figure 2**).

For the PMN-MDSC subgroups, the median survival durations were 159 and 768 days for the PMN-MDSC_low_ and PMN-MDSC_high_ subgroups, respectively. A log-rank test indicated a significant difference in survival durations between these two subgroups (P = 0.018) (**Figure 6B**). However, no statistically significant (P = 0.11) difference in survival was observed between the M-MDSC_low_ and M-MDSC_high_ subgroups, which suggests that PMN-MDSC, but not M-MDSC, might be a more critical marker for poor prognosis among patients with mCRPC (**Figure 6C**).

### 3.5 Association between the subtypes based on the MDSC levels and prognosis

To assess the prognostic significance of MDSC subtypes in patients with prostate cancer, we utilized the Cox proportional hazards models and explored influence of different variables on the risk associated with high PMN-MDSC levels. Each model was defined and adjusted as follows: Model 1 was age-adjusted to provide a HR that isolates the effect of MDSC levels from the age factor; Model 2 extends this and includes adjustments for age and BMI that offers insights into impact of MDSC levels on the prognosis independent of these common confounders; and Model 3, our most comprehensive model, which incorporates adjustments for age, BMI, presence of bone metastases, and chemotherapy treatments. This model was designed to evaluate the influence of MDSC in the context of multiple clinically relevant factors.

Our findings suggest that patients with high PMN-MDSC levels consistently showed poorer outcomes across all models, which highlights the robustness of the prognostic value of PMN-MDSC. The results are presented in **Table 2**, which showed that the univariate HR for PMN-MDSC_high_ patients was 3.41 (95% CI: 1.17–9.99, P = 0.025). The age-adjusted analysis (Model 1) yielded an HR of 3.64 (95% CI: 1.22–10.87, P = 0.021). In Model 2, adjusted for age and BMI, the HR was 4.62 (95% CI: 1.43–14.87, P = 0.010). Model 3, which further considered bone metastases and chemotherapy, showed an HR of 4.46 (95% CI: 1.34–14.85, P = 0.015).

**Table 2:** Prognostic factor analysis of MDSCs using univariate and multivariate Cox regression models.

Subsets of MDSC	Model	HR (95%CI)	P value
PMN-MDSC	univariate	3.41 (1.17–9.99)	0.025
	Model 1	3.64 (1.22–10.87)	0.021
	Model 2	4.62 (1.43–14.87)	0.01
	Model 3	4.46 (1.34–14.85)	0.015
M-MDSC	univariate	0.37 (0.10–1.33)	0.13
	Model 1	0.25 (0.06–1.00)	0.05
	Model 2	0.25 (0.06–1.02)	0.053
	Model 3	0.20 (0.04–0.99)	0.049

Model 1: adjusted with age group, Model 2: adjusted with age group and BMI group, Model 3: adjusted with age group, BMI group, chemotherapy and bone metastasis. CI, confidence interval; HR, hazard ratio; M-MDSC, monocytic myeloid-derived suppressor cell; PMN-MDSC, polymorphonuclear myeloid-derived suppressor cell.

Conversely, we observed a trend showing that higher M-MDSC levels might be associated with more favorable outcomes, although this finding was not statistically significant (**Table 2**). The univariate HR was 0.37 (95% CI: 0.10–1.33, P = 0.13). The age-adjusted HR of Model 1 was 0.25 (95% CI: 0.06–1.00, P = 0.050). Model 2, which was adjusted for age and BMI, obtained an HR of 0.25 (95% CI: 0.06–1.02, P = 0.053). Model 3, which was further adjusted, showed an HR of 0.20 (95% CI: 0.04–0.99, P = 0.049).

To further investigate the potential of PMN-MDSC and M-MDSC as prognostic factors, a sensitivity analysis was conducted by incorporating both factors into Model 3 (**Table 3**). Higher PMN-MDSC levels were significantly associated with poorer outcomes, with an HR of 3.48 (95% CI: 1.05–11.56, P = 0.042). Contrarily, M-MDSC was not associated with prognosis (HR, 0.24; 95% CI: 0.04–1.41, P = 0.11). Additionally, we performed an analysis to examine the interaction between PMN-MDSC and M-MDSC (**Table 4**), which revealed no significant interaction between the two factors (P = 0.50).

**Table 3:** Sensitivity analysis of the association between prognosis and MDSCs using multivariate Cox proportional hazards models.

Variables	HR (95%CI)	P value
PMN-MDSC	3.48 (1.05–11.56)	0.042
M-MDSC	0.24 (0.04–1.41)	0.11
Age	0.36 (0.07–1.74)	0.20
BMI	0.35 (0.05–2.62)	0.30
Bone metastasis	6.05 (0.47–78.59)	0.17
chemotherapy	3.25 (0.63–16.93)	0.16

BMI, Body Mass Index; CI, confidence interval; HR, hazard ratio; M-MDSC, monocytic myeloid-derived suppressor cell; PMN-MDSC, polymorphonuclear myeloid-derived suppressor cell.

**Table 4:** Multivariate Cox proportional hazards model including the interaction between PMN-MDSC and M-MDSC.

Variables	HR (95%CI)	P value
PMN-MDSC	4.50 (1.08–18.65)	0.038
M-MDSC	0.31 (0.05–2.07)	0.23
PMN-MDSC * M-MDSC	0.34 (0.02–7.56)	0.50
Age	0.32 (0.06–1.58)	0.16
BMI	0.25 (0.03–2.20)	0.21
Bone metastasis	8.26 (0.54–126.30)	0.13
Chemotherapy	4.22 (0.72–24.78)	0.11

BMI, Body Mass Index; CI, confidence interval; HR, hazard ratio; M-MDSC, monocytic myeloid-derived suppressor cell; PMN-MDSC, polymorphonuclear myeloid-derived suppressor cell.

## 4 Discussion

Our study examined the relationship between MDSCs and prostate cancer progression, focusing specifically on mCRPC. We found correlations between age, BMI, and M-MDSC levels in these patients; specifically, younger individuals and those with a higher BMI showed increased M-MDSC levels. In comparison to nmHSPC and mHSPC, the mCRPC was significantly increased in the PMN-MDSC. Additionally, to the best of our knowledge, this is the first report to identify an association between elevated PMN-MDSC levels and unfavorable outcomes in patients with mCRPC, highlighting the critical importance of MDSC measurement.

Aging has been recognized as a multifaceted process characterized by an increased accumulation of proinflammatory cytokines, concomitant with alterations in the composition and functionality of various immune cell types across the adaptive and innate immune spectra (26). The total MDSCs with age has been reported as a potential contributor to immunological abnormalities and pathologies observed in the elderly individuals (27). Verschoor et al. observed a significant increase in the frequency of total MDSCs and PMN-MDSCs in the elderly as compared to that observed in younger adults (28). Another study indicated that, although the total MDSC levels were higher in elderly individuals than in younger patients, the M-MDSC levels were significantly higher in the younger group, partially aligning with the findings of our study (29). This could imply that robust immune responses, perhaps more reactive to tumor antigens, might drive the compensatory upregulation of M-MDSCs as a mechanism to mitigate excessive inflammation in younger patients. Alternatively, the aggressive nature of tumors in younger individuals could directly and more profoundly stimulate M-MDSC expansion, reflecting a dynamic and aggressive tumor–immune interaction.

We observed elevated M-MDSC levels among the mCRPC patients with a higher BMI. Interestingly, this correlation was not evident with PMN-MDSC. This observation aligns with findings from previous studies that reported an association between increased BMI and higher M-MDSC levels, even in individuals without metabolic abnormalities (30). M-MDSCs were found to be expanded in obese/overweight Chinese men; however, the cohort size was very small, consisting of only eight normal controls and eight obese/overweight patients (30). In another study involving 27 normal-weight, 23 overweight, and 60 obese individuals, obese individuals were found to have higher M-MDSC levels (31). Evidence from mouse studies suggests that obesity-induced inflammation prompts macrophages to produce IL-6, resulting in the elevation of MDSC levels characterized by CD45^+^, CD11b^+^, Ly6G, and Ly6C^+^ markers (32). Additionally, hypoxic environments within tumors lead to IL-6 overexpression, specifically in malignant cells, resulting in the expansion of MDSCs expressed as Gr1^+^/CD11b^+^ in tumors (33). Adipose tissue, which is a characteristic of obesity, is known to foster chronic inflammation, potentially inducing an overproduction of leptin. This leptin overproduction can possibly stimulate the accumulation of PMN-MDSC and M-MDSC in the bloodstream and solid tumors (34). These obesity-induced PMN-MDSCs and M-MDSCs are implicated in suppressing tumor-reactive T cells and obstructing the entry of activated T cells into the TME, thereby promoting tumor proliferation. Furthermore, obesity in adulthood has been linked with worse outcomes among patients with prostate cancer, increasing their risk of developing advanced-stage disease, higher rates of recurrence, and greater cancer-specific mortality rates after diagnosis (35). A comprehensive meta-analysis has shown a 15% increase in the risk of fatal prostate cancer and 20% increase in prostate cancer-specific mortality for every 5-kg/m² increase in BMI (36). These findings suggest that the influence of obesity on prostate cancer prognosis may be mediated partly by its effect on the M-MDSC levels and function, further complicating the interplay between metabolic health and cancer progression.

Our findings indicate that the increase in the PMN-MDSC levels is more closely associated with the acquisition of hormonal resistance than with the presence or absence of metastasis. This insight advances beyond the findings a previous study, which primarily reported a link between PMN-MDSC levels and prostate cancer stage (19). Although the mechanisms underlying CRPC are not yet fully understood, PMN-MDSCs are known to be an important subset of immune cells that invade the CRPC microenvironment (37). Additionally, PMN-MDSC-derived exosomes increase the level of a molecule called circMID1 in prostate cancer cells via its specific protein (37). This elevation triggers a series of molecular interactions that contribute to CRPC progression. Another mechanism is that mCRPC have different genomic sequences and AR signaling pathway alterations as compared to HSPCs (38–41). These differences may suggest a unique response of mCRPC patients to immune changes associated with aging and BMI as compared to those with hormone-sensitive prostate cancer.

The most significant outcome of this research is the identification of an association between PMN-MDSC levels and prognosis in patients with mCRPC, supported by robust statistical evidence. Contrarily, decreased M-MDSC levels showed a potential association with an adverse outcome. This dynamic is potentially rooted in the differentiation of M-MDSC to PMN-MDSC, a process believed to be driven by an epigenetic change involving histone deacetylase 2 (HDAC-2) acting on the retinoblastoma gene (Rb1) (42). HDAC overexpression in prostate cancer, which is crucial for functional androgen receptor signaling (43), suggests their influential role in PMN-MDSC and M-MDSC dynamics within mCRPC. Our study aligns with emerging research highlighting MDSC’s critical role in prostate cancer progression. Specifically, MDSC-mediated IL-23 production bolsters castration resistance by preserving AR signaling (44). The possibility that mCRPC patients with low M-MDSCs is associated with a worse prognosis suggests that the proportion of M-MDSCs in the total PBMC population may be relatively reduced because of progressive differentiation into PMN-MDSCs in the castration-resistant state. However, this association between M-MDSC and prognosis was not statistically significance in our analysis. Additionally, the sensitivity analysis did not reveal any interaction between PMN-MDSC and M-MDSC. Consequently, a future study involving a larger sample size may be required to elucidate the dynamics more comprehensively between M-MDSC and PMN-MDSC.

M-MDSCs are characterized by their high suppressive activity, primarily through nitric oxide (NO) production via the inducible nitric oxide synthase (iNOS) (45–47). Contrarily, PMN-MDSCs exert their immunosuppressive effects through different pathways, primarily involving reactive oxygen species (ROS) and peroxynitrite (PNT) productions, mediated by enzymes like nox2 and endothelial NO synthase (nos3) (48). The association of elevated PMN-MDSC levels with poorer outcomes among mCRPC patients underscores the evolving and complex interplay of these cell types in cancer immunology and progression. A previous study identified significant infiltration of PMN-MDSCs, particularly in the stromal compartments of primary and metastatic prostate cancers, with a greater infiltration noted in the stromal areas of the metastatic sites than in the primary tumors (49). These findings underscore the role of the stroma as a significant reservoir for PMN-MDSCs, supporting their involvement in promoting vascularization, immune evasion, and possibly metastatic progression of prostate cancer. In mCRPCs, where patients often exhibit advanced disease characterized by high tumor burden and immune evasion, circulating PMN-MDSCs may serve as a herald of heightened immunosuppressive activity within tumors. The infiltration patterns described in the previous study suggest that these cells, once recruited to the tumor stroma, contribute significantly to the creation of an immunosuppressive niche that protects tumor cells from immune surveillance and facilitates tumor growth and metastasis (49).

Our study addresses several critical gaps identified in the current MDSC research in prostate cancer, particularly mCRPC. Although previous studies have highlighted significant limitations due to model fidelity, immune system discrepancies between species, technical challenges in MDSC profiling, and biological heterogeneity of prostate cancer, our research implemented robust methodologies to overcome these challenges and provide meaningful insights into the role of MDSCs in mCRPC progression (50). Additionally, recognizing the technical issues, including the impact of cryopreservation on MDSC phenotyping and functional assays, our study strictly utilized fresh blood samples, which were processed within 4 hours of collection (24). This approach ensures the reliability of our study findings related to MDSC phenotypes and functions, addressing the concerns raised about the potential biases introduced by sample handling and preservation. As previous studies have reported the need for more homogeneous patient groups, this study carefully selected participants based on well-defined criteria of disease state, from nmHSPC and mHSPC to mCRPC. This stratification allows for a meticulous understanding of MDSC dynamics across different stages of prostate cancer progression, enhancing the generalizability of our findings within each specific context.

However, our study presents several limitations. First, the causative relationship between elevated MDSC levels in mCRPC patients, as compared to mHSPC patients, remains unclear. Our analysis was constrained due to the limited number of included mHSPC patients who transitioned to mCRPC within our study duration. Second, mCRPC patients have undergone various treatment lines, making it impossible to standardize the baseline. Therefore, although we adjusted for confounding factors through a multivariate analysis, further research is necessary to determine the appropriate timing for MDSC measurement. Third, sample size determination in our study was based on consecutive inclusion within a predefined time frame rather than being calculated explicitly for statistical power. Thus, the absence of random sampling or randomization may introduce a selection bias, potentially impacting the validity and reliability of our findings. Additionally, the role of M-MDSCs in prostate cancer progression and prognosis is also complex. Despite observing a trend wherein higher M-MDSC levels might be associated with more favorable outcomes, these findings were not statistically significant; hence, it should be interpreted with caution. This highlights a potential limitation in the predictive value of M-MDSCs within our study cohort and underscores the need for further investigation in the future to clarify their biological impact and clinical utility in different stages of prostate cancer. Such studies could help in establishing the potential of M-MDSC levels as a reliable biomarker for determining prognosis or reflecting other underlying biological processes in the tumor microenvironment.

In conclusion, our study illuminates an association between PMN-MDSC levels and prognosis in patients with mCRPC and underscores the higher proportion of MDSCs in those with mCRPC than those with mHSPC. Further validation is required to see if the association can be replicated in other institutions and populations.

## Data availability statement

The raw data supporting the conclusions of this article will be made available by the authors, without undue reservation.

## Ethics statement

The studies involving humans were approved by the Juntendo University Institutional Review Board (protocol code: M19-0158 and H20-0187, date of approval: Nov. 1, 2019 and Sep. 11, 2020). The studies were conducted in accordance with the local legislation and institutional requirements. The participants provided their written informed consent to participate in this study.

## Author contributions

TK: Writing – original draft, Conceptualization, Formal analysis, Funding acquisition, Methodology, Software, Visualization, Writing – review & editing. MN: Conceptualization, Supervision, Writing – review & editing. TH: Investigation, Writing – review & editing. HW: Data curation, Investigation, Resources, Writing – review & editing. YI: Data curation, Formal analysis, Software, Validation, Writing – review & editing. KT: Data curation, Writing – review & editing. JL: Resources, Writing – review & editing. YJ: Resources, Writing – review & editing. YL: Formal analysis, Methodology, Resources, Software, Writing – review & editing. TC: Writing – review & editing. FS: Writing – review & editing, Supervision. HI: Supervision, Writing – review & editing. SH: Supervision, Writing – review & editing.
