# Supplementary material for: Increased circulating polymorphonuclear myeloid-derived suppressor cells are associated with prognosis of metastatic castration-resistant prostate cancer
Source: Front Immunol. 2024 Jun 3;15:1372771. doi: 10.3389/fimmu.2024.1372771 (PMC11180772; doi:10.3389/fimmu.2024.1372771)
Supplement: Supplementary file 1 [file Image_1.pdf]

Supplementary Material

1 Supplementary Figures

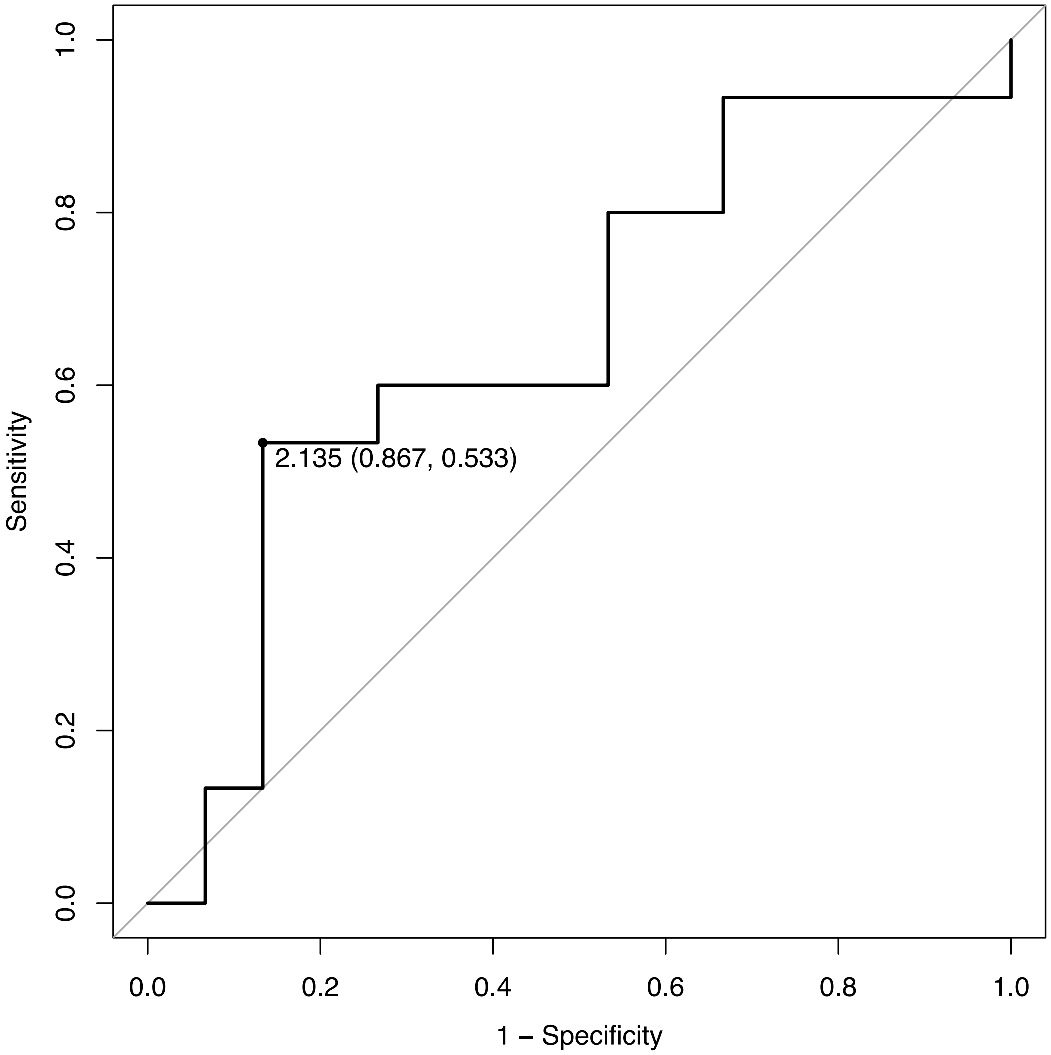

Supplementary Figure 1. Receiving-operating characteristic curve of PMN-MDSC. The cutoff value of PMN-MDSC (2.135) was defined by using the Youden index. PMN-MDSC, polymorphonuclear myeloid-derived suppressor cell

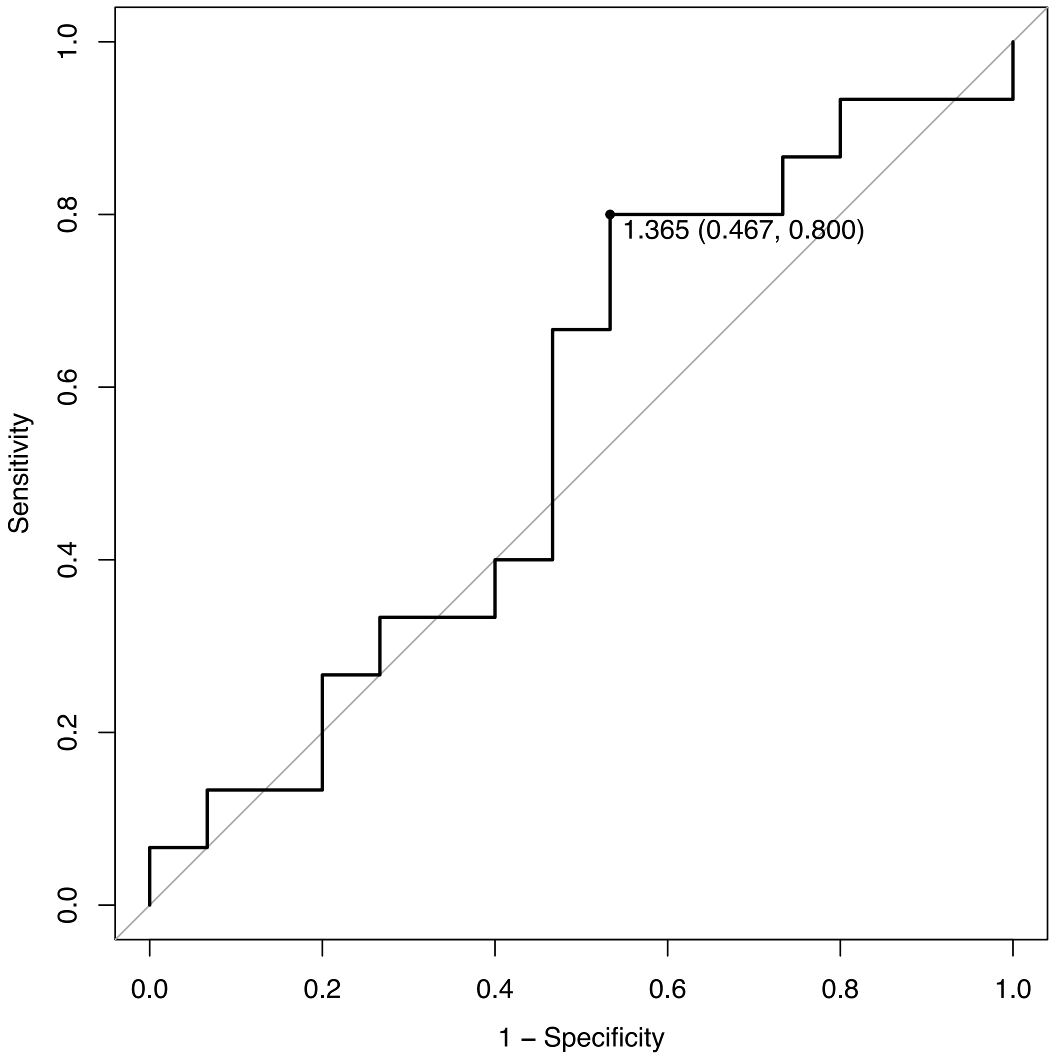

Supplementary Figure 2. Receiver-operating characteristic curve of M-MDSC. The cutoff value of M-MDSC (1.365) was defined by using the Youden index. M-MDSC, monocytic myeloid-derived suppressor cell
